# Characterizing Tau Accumulation and Amyloid‐Tau Interactions in the Alzheimer’s Prevention Initiative Autosomal Dominant Alzheimer’s Disease Colombia Trial and Their Implications for Advancing Drug Development

**DOI:** 10.1002/alz70859_100956

**Published:** 2025-12-25

**Authors:** Nicolo Foppa Pedretti, Yashmin Karten, Yi Zhang, Gregory Klein, Pierre N. Tariot, Philip S. Insel, Yi Su, Robert C. Alexander, Eric M. Reiman, Nadine Tatton, Critical Path for Alzheimer's Disease (CPAD) Consortium

**Affiliations:** ^1^ Critical Path Institute, Tucson, AZ USA; ^2^ F. Hoffmann‐La Roche Ltd, Basel Switzerland; ^3^ Banner Alzheimer's Institute, Phoenix, AZ USA; ^4^ University of California, San Francisco, San Francisco, CA USA

## Abstract

**Background:**

Alzheimer’s disease (AD), particularly its autosomal dominant form (ADAD), presents unique challenges for drug development. Clinical trials involving cognitively healthy individuals with ADAD can provide a useful model for sporadic Alzheimer's disease. They can aid in studying the early stages of the disease, which is challenging in sporadic cases due to heterogeneity. The CPAD consortium integrates clinical trial and observational data to develop tools to accelerate drug development. A key goal is to create a comprehensive drug‐disease‐trial model for ADAD, using data from multiple sources to address critical questions on tau and amyloid pathologies, biomarker‐cognition relationships, and disease progression.

**Methods:**

The Alzheimer’s Prevention Initiative ADAD Colombia Trial1 is a placebo‐controlled clinical trial of crenezumab in 252 cognitively unimpaired Presenilin 1 (PSEN1) E280A kindred members. Initial research questions guiding this study include: (1) evaluating regional tau accumulation patterns in ADAD compared to sporadic AD to determine whether the same brain regions show the fastest tau accumulation; and (2) estimating the association between changes in regional tau and amyloid PET to assess whether local amyloid‐tau relationships within regions are stronger than across‐brain correlations. We use a linear mixed effect model to evaluate longitudinal outcomes with a model specification including fixed effects for mutation status, time, interaction terms, and random effects structured to account for individual heterogeneity. Model comparison techniques are used to select the most parsimonious random effects structure, focused on regional tau accumulation patterns and amyloid‐tau relationship dynamics across brain regions.

**Results:**

Linear mixed effect models revealed distinct tau accumulation patterns in carriers compared to non‐carriers. Regional tau protein accumulation differed across brain regions, with specific variations in baseline amyloid‐related dynamics and demographic effects. The analysis distinguished cognitive progression characteristics between ADAD and sporadic AD, highlighting longitudinal neurodegenerative mechanisms.

**Conclusion:**

We characterize distinct tau accumulation patterns and amyloid‐tau interactions, particularly in ADAD mutation carriers, offering valuable insights into early disease progression. We tested our model assumptions and methods, further strengthening the framework for advancing therapeutic research and understanding the neurodegenerative mechanisms underlying both ADAD and sporadic AD.

